# Protective Effects of Silymarin and Silibinin against DNA Damage in Human Blood Cells

**DOI:** 10.1155/2018/6056948

**Published:** 2018-10-02

**Authors:** Flávio Fernandes Veloso Borges, Carolina Ribeiro e Silva, Wanessa Moreira Goes, Fernanda Ribeiro Godoy, Fernanda Craveiro Franco, Jefferson Hollanda Véras, Elisa Flávia Luiz Cardoso Bailão, Daniela de Melo e Silva, Clever Gomes Cardoso, Aparecido Divino da Cruz, Lee Chen-Chen

**Affiliations:** ^1^Laboratório de Radiobiologia e Mutagênese, Instituto de Ciências Biológicas, Universidade Federal de Goiás, Campus II, Goiânia, GO, Brazil; ^2^Laboratório de Mutagênese (LABMUT), Instituto de Ciências Biológicas, Universidade Federal de Goiás, Campus II, Goiânia, GO, Brazil; ^3^Laboratório de Biotecnologia, Câmpus Henrique Santillo, Universidade Estadual de Goiás, Anápolis, GO, Brazil; ^4^Núcleo de Pesquisas Replicon, Escola de Ciências Agrárias e Biológicas, Pontifícia Universidade Católica de Goiás, Goiânia, GO, Brazil

## Abstract

Silymarin (SM), a standardized extract derived from* Silybum marianum* (L.) Gaertn, is primarily composed of flavonolignans, with silibinin (SB) as its major active constituent. The present study aimed to evaluate the antigenotoxic activities of SM and SB using the alkaline comet assay in whole blood cells and to assess their effects on the expression of genes associated with carcinogenesis and chemopreventive processes. Different concentrations of SM or SB (1.0, 2.5, 5.0, and 7.5 mg/ml) were used in combination with the DNA damage-inducing agent methyl methanesulfonate (MMS, 800 *μ*M) to evaluate their genoprotective potential. To investigate the role of SM and SB in modulating gene expression, we performed quantitative real-time PCR (qRT-PCR) analysis of five genes that are known to be involved in DNA damage, carcinogenesis, and/or chemopreventive mechanisms. Treatment with SM or SB was found to significantly reduce the genotoxicity of MMS, upregulate the expression of* PTEN* and* BCL2*, and downregulate the expression of* BAX *and* ABL1*. We observed no significant changes in* ETV6 *expression levels following treatment with SM or SB. In conclusion, both SM and SB exerted antigenotoxic activities and modulated the expression of genes related to cell protection against DNA damage.

## 1. Introduction

According to the World Health Organization, 70% to 95% of the world's population rely on traditional medicine for primary health care, and most health practices involve the use of plant extracts or their active components [1].* Silybum marianum* (L.) Gaertn, popularly known as milk thistle, is one of the most widely used herbs worldwide.* S. marianum* has been well-known since ancient times and has been mostly used in traditional European and Asian medicine for treatment of liver disorders [2].

The medicinal properties of* S. marianum* are attributed to its ability to accumulate bioactive flavonolignan complexes, which are referred to as silymarin (SM).* S. marianum* contains approximately 70% to 80% flavonolignans (silymarin complex), small amounts of flavonoids, 20% to 30% fatty acids, and other polyphenolic compounds. The flavonolignan mixture present in* S. marianum* mainly consists of silibin (SB), also known as silibinin, the major bioactive component of the extract. Milk thistle extract is currently marketed worldwide as silymarin and silibinin under various trade names, such as Siliphos, Silipide, and Legalon [3].

The effects of SM and SB have been investigated in mice, rats, rabbits, and dogs and results demonstrated that their acute, subacute, and chronic toxicities are very low. SM and SB are primarily used for the treatment of various liver disorders that are characterized by degenerative necrosis and functional impairment, such as chronic inflammatory diseases, cirrhosis, and toxic liver damage [4]. In addition, SM and SB are well documented to exhibit various biological and pharmacological activities, including antioxidant [5], antidiabetic [6], anti-inflammatory, and immunomodulatory effects [7].

The pharmacological activities and toxicological safety of SM and SB have been extensively studied both* in vitro* and* in vivo*. However, little is known about their protective effects on the genetic material. Numerous phytochemicals have been reported to interfere with specific stages of carcinogenesis, and multiple mechanisms have been shown to account for the anticarcinogenic properties of dietary constituents [8].

Analysis of genes related to DNA damage, carcinogenesis, and/or chemoprevention can help elucidate the mechanisms by which dietary supplements can exert protective effects on DNA [9–13]. Thus, given the limited knowledge of the chemopreventive effects of SM and SB, analysis of the expression patterns of the tumor suppressors genes* ETV6* and* PTEN*, the cell death regulators* BCL2* and* BAX* (pro/antiapoptotic processes), and the protooncogene* ABL1* can reveal the molecular basis underlying the effects of SM and SB.

Considering the biological activities presented by SM and SB, as well as their widespread use as herbal medicines, the present study aimed to evaluate the antigenotoxic activities of SM and SB using the comet assay and to evaluate the expression patterns of genes that are known to be associated with carcinogenesis and chemopreventive processes.

## 2. Material and Methods

### 2.1. Chemicals

Silymarin (SM, S0292), silibinin (SB, S0417), RPMI 1640 medium, methyl methanesulfonate (MMS), ethidium bromide, dimethyl sulfoxide (DMSO), NaCl, and Triton X-100 were purchased from Sigma-Aldrich (St. Louis, MO, USA). Low melting point agarose and normal melting point agarose were obtained from Thermo Fisher Scientific (Waltham, MA, USA). Na_2_EDTA, Tris base, and Tris-HCl were purchased from Bio-Rad Laboratories (Hercules, CA, USA).

### 2.2. Cell Treatment

Peripheral blood was obtained through venous puncture from three young and healthy volunteers who had no history of smoking or drinking. Our work was approved by the Human and Animal Research Ethics Committee of the Universidade Federal de Goiás (CEPMHA/HC/UFG n. 016/2011). Whole blood was treated with varying concentrations of SM or SB (1.0, 2.5, 5.0, and 7.5 mg/ml) in combination with 800 *μ*M MMS and subsequently incubated for 3 h at 37°C and 5% CO_2_ in RPMI medium containing 15% (v/v) fetal calf serum. The positive (MMS) and negative (DMSO) control groups were included. The experiment was performed in triplicate. The MMS concentration of 800 *μ*M used in the present study was selected based on its previously demonstrated effectiveness in inducing DNA damage [17].

### 2.3. Comet Assay

The alkaline version of the comet assay was performed according to the protocol described by Singh and coworkers [18] with slight modifications. For antigenotoxicity evaluation, cells were treated with SM and SB, concomitant with the positive control, in order to verify the possible reduction of DNA damage caused by MMS. Briefly, after cell treatment, slides coated with normal melting point agarose (1.5%) were added with a mixture containing 15 *μ*l of blood and 130 *μ*l of low melting point agarose (0.5%) and incubated at 37°C. The mixture was spread on the slides with coverslips and placed in a cold chamber. Afterwards, the coverslips were carefully removed, and the slides were immersed in lysis solution protected from light (1% Triton X-100, 10% DMSO, 2.5 M NaCl, 100 mM EDTA-Na_2_, and 10 mM Tris) at 4°C for 4 h. Subsequently, the slides were incubated with freshly prepared alkaline solution buffer (300 mM NaOH and 1 mM EDTA, pH > 13) at 4°C for 20 min to unwind the DNA. Samples were then subjected to electrophoresis in the same buffer at 1 V/cm and the current of 300 mA for 30 min in the dark. After electrophoresis, the slides were placed on a staining tray, covered with neutralizing buffer (0.4 M Tris-HCl, pH 7.5), and kept in the dark for 5 min. For analysis, the slides were stained with 20 *μ*l of ethidium bromide solution (0.02 mg/ml) and covered with a cover slip. A total of 50 nucleoids were analyzed per slide, corresponding to 100 nucleoids per sample.

The analysis was performed on a fluorescence microscopy system Axioplan-Imaging® (Carl Zeiss AG, Germany) using the Isis software with an excitation filter of 510-560 nm and a barrier filter of 590 nm under 20× magnification. To assess genomic damage, we used the TriTek Comet ScoreTM software (version 1.5), in which pixels intensities are used to estimate the degree of genomic damage and are given as arbitrary units. The nucleoids with completely fragmented heads were not included in the analysis.

From the 17 parameters provided by the software, we selected the percentage of DNA in the tail for assessing DNA damage. This parameter has been proposed by several authors to be the most useful parameter because it provides a quantitative measure of DNA damage (from 0 to 100%) [19].

### 2.4. Calculation of DNA Damage Reduction Percentage in Comet Test

For antigenotoxicity assessment, the percentage of reduction in MMS-induced damage by SM and SB was calculated according to Waters et al., 1990 [20], using the following formula:

(1) $$\begin{array}{c} \%\;\;Reduction=\left(\;\frac{\left(A-B\right)}{\left(A-C\right)}\;\right)\times 100 \end{array}$$

where A corresponds to the DNA damage observed following treatment with MMS (positive control), B represents the group treated with SM or SB plus MMS, and C represents the negative control.

### 2.5. RNA Extraction and cDNA Synthesis

After treatment, human blood samples were transferred to tubes provided by GeneJET™ Whole Blood RNA Purification Mini Kit (Thermo Fisher Scientific, Inc., USA). RNA extraction was performed according to the manufacturer's instructions. Afterwards, the final RNA concentration was determined using a spectrophotometer NanoVuePlusTM (GE Healthcare, USA). RNA purity and integrity were assessed via 3% agarose gel electrophoresis. To ensure the purity of the RNA, the A260/A230 and A260/A280 absorbance ratios were evaluated. cDNA synthesis was performed using 1 *μ*g of total RNA in a 10 *μ*L sample volume using RT2 First Strand Kit® (PreAnalytix QIAGEN/BD Company, Germany) as recommended by the manufacturer. Amplified cDNA was stored at -20°C.

### 2.6. Quantitative Real-Time PCR (qRT-PCR) Design and Test

Customized qRT-PCR assay was performed using 96-well-plates. We analyzed five target genes (*ETV6*,* PTEN*,* ABL1*,* BAX*, and* BCL2*) using* GAPDH* as reference gene. In addition, the plates contained a genomic DNA control, a reverse-transcription control, and a positive PCR control.

The qRT-PCR using cDNA derived from treated human whole blood cells was performed using the RT2 SYBR Green Master Mix Kit® (PreAnalytix QIAGEN/BD Company, Germany) according to the manufacturer's instructions. Array-based qRT-PCR analysis was performed in 28 *μ*L reaction volumes containing 1 *μ*L of cDNA and 14 *μ*L of RT2 SYBR Green Master Mix. Subsequently, 25 *μ*L of PCR mix was added to each well of the RT2 Profiler PCR Array. Reactions were run on Bio-Rad's IQ5 real-time thermal cycler using the following cycling conditions: 1 cycle at 95°C for 10 min; 40 cycles of 15 s at 95°C; and 1 min at 60°C. The melting curve was performed as follows: 1 cycle of 1 min at 95°C, 2 min at 65°C, and 2 min at 65°C for 95°C. Results were obtained using iQ5® Optical System software version 2.1 and exported using Microsoft Excel (Microsoft Corporation, USA). Gene expression analysis was carried out using the comparative ΔΔCt method.

The cycle threshold (Ct) values were exported to the PCR Array Data Analysis Web Portal (http://dataanalysis.sabiosciences.com/pcr/arrayanalysis.php). First, gene expression levels for each sample were normalized against those of the reference gene* GAPDH* (ΔCt). Ct data was used as an input, and the web-based software will automatically perform quantification using the ΔΔCt method (using positive control MMS treatment as standard sample). The fold change was calculated for each gene for all group samples.

### 2.7. Statistical Analysis

For the comet assay, treatment and control groups were analyzed by performing one-way ANOVA, followed by Tukey's test. Statistical significance was considered at* P* < 0.05. All statistical analyses were conducted using GraphPad Prism 5.0.

For gene expression analysis via qRT-PCR, gene expression levels corresponding to each cotreatment (positive control + silymarin or silibinin) were compared relative to the positive control (MMS) by performing the Student's* t*-test. The fold change values were calculated using the ΔΔCt method. A fold change > 2.0 or* P* < 0.05 was considered significant.

## 3. Results

### 3.1. Modulation of MMS-Induced DNA Damage by Silymarin and Silibinin

**Figure 1:**
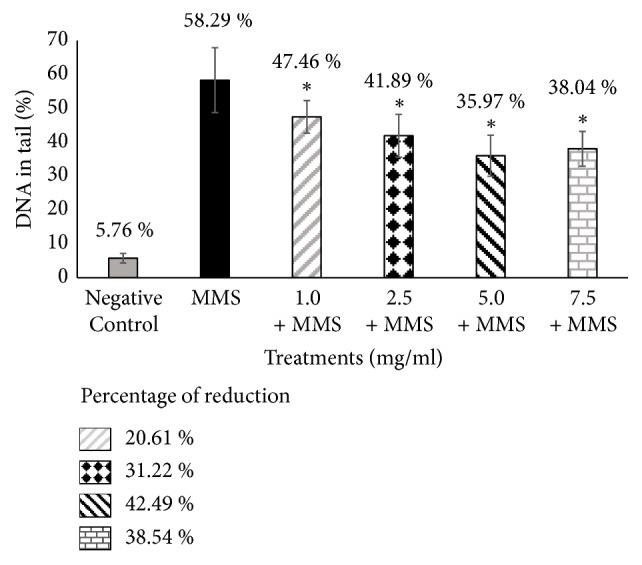
**Evaluation of the antigenotoxic effects of silymarin by comet assay.** Results are expressed as mean ± standard deviation (SD). Percentage reduction in MMS-induced damage by SM. Negative control: 100 *µ*L of dimethylsulfoxide (DMSO) + sterile distilled water (1:1). Positive control: methyl methanesulfonate (MMS) (800 *µ*M). *∗ P* < 0.05 versus MMS.

**Figure 2:**
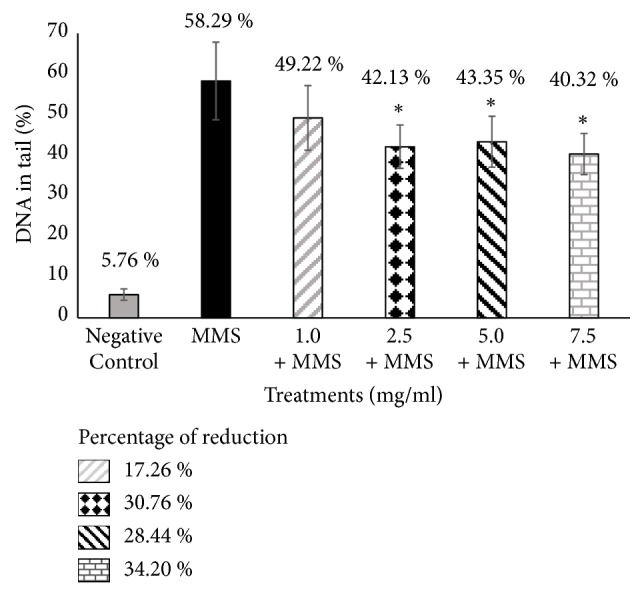
**Evaluation of the antigenotoxic effects of silibinin by the comet assay.** Results are expressed as mean ± standard deviation (SD). Percentage reduction in MMS-induced damage by SB. Negative control: 100 *µ*L of dimethylsulfoxide (DMSO) + sterile distilled water (1:1). Positive control: methyl methanesulfonate (MMS) (800 *µ*M). *∗ P* < 0.05 versus MMS.

Assessment of the antigenotoxicity of SM and SB via the alkaline comet assay demonstrated reduced DNA damage (% DNA in tail) in cells cotreated with SM or SB and MMS relative to cells treated with the positive control MMS alone (Figures 1 and 2).

Treatment with the standardized extract (SM) significantly reduced DNA damage for all tested concentrations of the compound when combined with MMS relative to treatment with MMS alone. SB, the major active constituent of SM, also exerted significant protective effect against DNA damage induced by the genotoxic agent MMS, except at a lower concentration (1.0 mg/ml) (Figures 1 and 2). The percentage of DNA in tail ranged from 47.46% to 38.04% for SM with MMS and 49.22% to 40.32% for SB with MMS. The percent reduction in DNA damage ranged from 20.61% to 38.54% for SM with MMS and 17.26% to 34.20% for SB with MMS when compared to treatment with MMS alone (58.29% DNA in tail).

### 3.2. Effects of Silymarin and Silibinin in Combination with MMS on Gene Expression in Human Blood Cells

We evaluated the expression levels of the following five genes associated with DNA damage, carcinogenesis, and/or chemoprevention mechanisms: the tumor suppressors* ETV6* and* PTEN*, the cell death regulators* BCL2* and* BAX* (anti- and proapoptotic, respectively), and the protooncogene* ABL1*.

**Figure 3:**
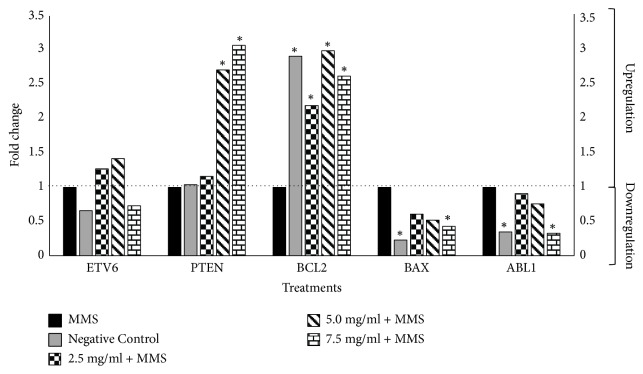
**Effects of combined treatment with MMS and silymarin on gene expression relative to MMS alone.** Positive control: methyl methanesulfonate (MMS) (800 *µ*M). Negative control: 100 *µ*L of dimethylsulfoxide (DMSO) + sterile distilled water (1:1). Expression values greater than one indicate an upregulation, while expression values less than one indicate downregulation in the test sample relative to the positive control. *∗ P* < 0.05 versus MMS.

**Figure 4:**
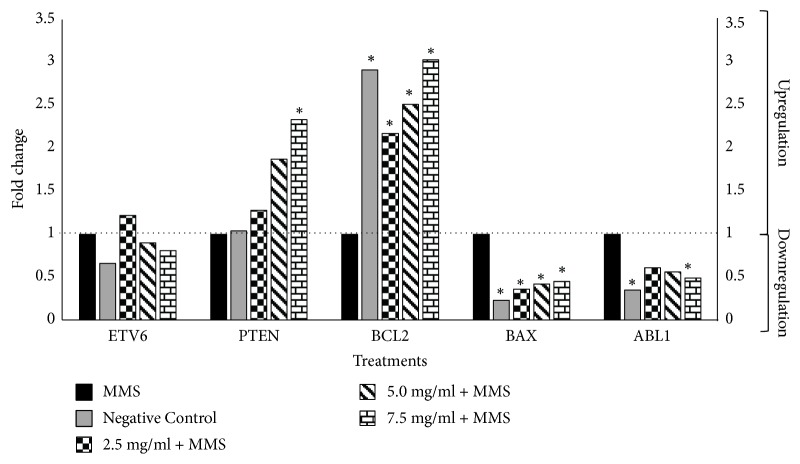
**Effects of combined treatment with MMS and silibinin on gene expression relative to MMS alone. **Positive control: methyl methanesulfonate (MMS) (800 *µ*M). Negative control: 100 *µ*L of dimethylsulfoxide (DMSO) + sterile distilled water (1:1). Expression values greater than one indicate upregulation, while expression values less than one indicate downregulation in the test sample relative to the positive control. *∗ P* < 0.05 versus MMS.

Expression levels of the tumor suppressor* ETV6* gene (Figures 3 and 4) were not significantly altered in all samples treated with varying concentrations of SM + MMS or SB + MMS relative to those treated with MMS alone. However, results showed that the expression levels of the tumor suppressor gene* PTEN* were significantly upregulated following cotreatment with high SM concentrations (5.0 and 7.5 mg/ml) and the highest SB concentration (7.5 mg/ml), corresponding to fold change values of 2.71, 3.07, and 2.33, respectively (Figures 3 and 4).

Treatment with SM and SB at all concentrations was found to upregulate the expression of the antiapoptotic gene* BCL2* up to threefold relative to the positive control, similar to the expression values obtained for the negative control. In addition, expression of the proapoptotic gene* BAX* was significantly downregulated following treatment with SM at the highest concentration and SB at all tested concentrations (Figures 3 and 4).

Furthermore, results demonstrated that the expression of* ABL1*, an apoptosis promoter and cell growth inhibitor gene, was significantly downregulated when human whole blood cells were treated with the highest concentration of SB or SM (7.5 mg/ml) (Figures 3 and 4).

## 4. Discussion

Many antioxidants are known to inhibit DNA damage by scavenging reactive oxygen species (ROS) that are generated inside the cell [21]. Several plant species have been reported as reliable sources of antioxidants, and multiple studies have demonstrated that plant compounds promote genomic stability through various mechanisms [11–13, 22]. Silymarin (SM) and silibinin (SB) are known to exhibit strong antioxidant activities [5], and their protective effects against ROS have been demonstrated using different cell types, including mouse lymphocytes and human platelets [16, 23–25]. Furthermore, treatment with SM and SB was found to enhance the activity of endogenous antioxidant enzymes, including glutathione peroxidase [26], which in turn inhibits ROS production. Therefore, the present study aimed to evaluate the antigenotoxic activities of SM and SB using the comet assay and to assess their effects on the expression pattern of genes associated with carcinogenesis and chemopreventive processes.

To evaluate the chemopreventive effects of SB and SM on the DNA, human whole blood cells were treated with SB or SM in combination with methyl methanesulfonate (MMS). MMS is an alkylating agent that induces damage to genetic material and forms monoadducts with the nucleophilic centers of DNA [27]. Damage to the genetic material is highly associated with enhanced ROS production, as well as methylation at the N-7 position of guanine (N7MeG), at the N-3 position of adenine (N3MeA), and at the O-6 position of guanine (O6MeG) [14, 15]. A previous study on human lymphocytes and sperm cells indicated that MMS exposure promoted DNA damage based on the comet assay (increased Olive Tail Moment and % DNA in tail) and triggered the apoptotic response by upregulating the expression of* TP53* and* CDKN1A* and downregulating the expression of* BCL2* [28].

Our results showed that both the complex (SM extract) and its main active constituent (SB), cotreated with MMS, exerted protective effects by reducing the amount of DNA in the comet tail in 42.49% and 34.20% respectively, when compared to positive control (MMS). Previous studies also demonstrated the protective effects of SM and SB. In particular, SM and SB significantly decreased point mutations based on the Ames test and reduced the proportion of micronucleated polychromatic erythrocytes based on the mice bone marrow assay [29]. Furthermore, SB was demonstrated to exert protective effects against *γ*-radiation-induced strand breaks in plasmid DNA, reduce DNA damage and micronuclei formation in human lymphocytes and rat leukocytes, and reduce mouse mortality and DNA damage in blood leukocytes following whole-body *γ*-exposure in mice [16, 30].

In addition, the current findings revealed that the extract complex (SM) exerted slightly stronger antigenotoxic activity compared to the primary active constituent (SB); however, the observed difference was not statistically significant. Our current findings were consistent with those of a previous study, which demonstrated that “high purity” milk thistle extracts exerted weaker antioxidant activity relative to the complex extract [29]. The above results suggest that the SM extract contains compounds other than SB that contribute to the antioxidant potential of SM. The final response of a treatment with a plant extract is a result of synergistic, antagonistic, and other interactive effects among plant biologically active compounds present in the extract and the cell machinery [31].

To elucidate the effects of cotreatment with MMS and SM or SB, we evaluated the expression levels of five genes that are specifically known to mediate chemoprevention and response to DNA damage.

The tumor suppressor gene* ETV6* (ets variant gene 6) encodes a protein that functions as a transcriptional regulator by binding to a specific DNA sequence. Our current findings revealed that the* ETV6* expression patterns were not significantly altered following treatment with any tested concentration of SM and SB when compared to the positive control, indicating that SM and SB did not influence* ETV6* expression.

The* PTEN* (phosphatase and tensin homolog) gene is a tumor suppressor involved in cell migration and proliferation inhibition and participates in the modulation of cell growth and apoptosis. The essential role of nuclear PTEN in maintaining chromosomal stability has been demonstrated in both mouse and human systems [32]. According to Yin and Shen [33], nuclear PTEN may utilize two mechanisms to maintain chromosome integrity. First, PTEN interacts with centromeres and maintains their stability through its C2 domain. Second, PTEN may be necessary for DNA repair, since loss of* PTEN* leads to a high proportion of double-strand breaks. Our results demonstrated significant upregulation of* PTEN* expression following treatment with high concentrations of SM or SB, what could contribute to explain the anticytotoxic and antigenotoxic properties of SM and SB. Also, these data allow us to infer that these protective effects may also help in the regulation and preservation of DNA in cells that are in the active process of cell division as shown in a study using mice bone marrow cells, in which SM and SB reduced the frequency of micronucleated cells [29].

The Bcl-2 family comprises proapoptotic and antiapoptotic proteins. BCL2 (B-cell lymphoma protein 2) is associated with programmed cell death inhibition in various cell types. The antiapoptotic function of BCL2 appears to be mediated by its ability to heterodimerize with other Bcl-2 family members, especially BAX (BCL-2 associated X protein). BCL2 prevents the oligomerization of BAX, which normally causes the release of several mitochondrial apoptogenic molecules [34]. Our results showed that treatment with SM or SB significantly upregulated* BCL2* expression at all tested concentrations. MMS is known to downregulate* BCL2* expression [15]; however, SM and SB can upregulate the expression of this survival factor by interacting with the* BCL2* promoter, which harbors several putative responsive sites, or through an indirect pathway. In addition, treatment with SM or SB significantly downregulated the expression of* BAX*, thereby suggesting that SM and SB can prevent apoptosis and act as chemoprotective agents. Previous studies demonstrated the modulatory effects of SM and SB on cell survival and apoptosis via interference with the expression of cell cycle regulators and proteins involved in apoptosis [6, 35–37].

The* ABL1* gene is a protooncogene that encodes a protein tyrosine kinase known to be involved in various cellular processes, including cell division, adhesion, differentiation, and stress response. Nuclear ABL proteins modulate cellular responses induced by DNA damage and are known to participate in cell growth inhibition and apoptosis promotion [38]. In the present study, treatment with the highest tested concentration of SM and SB was found to significantly downregulate* ABL1* expression relative to treatment with MMS alone, although the lower SM and SB concentrations did not significantly alter* ABL1* expression patterns. The observed downregulation of* ABL1* expression can be associated with the repair of DNA lesions.

**Figure 5:**
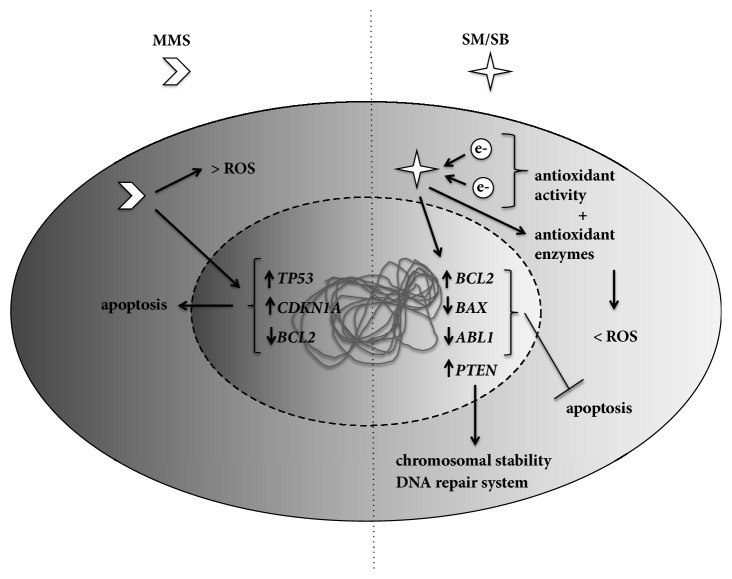
**Schematic representation of the effects of silymarin and silibinin on chemopreventive mechanisms.** While methyl methanesulfonate (MMS) is highly associated with enhanced reactive oxygen species (ROS) production [14] and apoptotic response [15], silymarin (SM) and silibinin (SB) exhibit strong antioxidant activities [5, 16] and can prevent apoptosis and influence the genomic stability and the DNA repair system.

The decrease in DNA damage and the modulation of gene expression to protect cells against lesions suggested the roles of SM and SB in the DNA repair system (Figure 5). Thus, the protective effects of both compounds highlighted their potential clinical use as complementary treatment for cancer patients in combination with established treatments to prevent or reduce the toxicity induced by chemotherapy and/or radiotherapy. However, further studies are required to investigate the effects of SM and SB on the DNA repair system.

## Data Availability

The data used to support the findings of this study are included within the article.
